# pH-Sensitive Glycyrrhizin Based Vesicles for Nifedipine Delivery

**DOI:** 10.3390/molecules26051270

**Published:** 2021-02-26

**Authors:** Olga Yu. Selyutina, Anna V. Mastova, Ekaterina A. Shelepova, Nikolay E. Polyakov

**Affiliations:** 1 Institute of Chemical Kinetics and Combustion, Institutskaya St. 3, 630090 Novosibirsk, Russia; mastova-anna99@yandex.ru (A.V.M.); shcat.95@mail.ru (E.A.S.); polyakov@kinetics.nsc.ru (N.E.P.); 2 Institute of Solid State Chemistry and Mechanochemistry, Kutateladze St. 18, 630128 Novosibirsk, Russia

**Keywords:** nifedipine, glycyrrhizin, pH-controlled drug delivery systems, inclusion complexes, NMR, molecular dynamics

## Abstract

Glycyrrhizic acid, or glycyrrhizin (GA), a major active component of licorice root, has been widely used in traditional Chinese and Japanese medicine since ancient times. However, only in the last decades has a novel and unusual property of the GA been discovered to form water-soluble, supramolecular complexes with a variety of lipophilic drugs. These complexes show significant advantages over other known delivery systems, in particular, due to strong pH sensitivity, the properties of GA self-associates. In the present study, a supramolecular complex formation of the hypotensive and antiarrhythmic drug nifedipine with GA has been studied at different pH values, corresponding to the different degrees of GA dissociation, including a fully dissociated state of GA. Both NMR experiments and molecular dynamics simulations demonstrate the existence of the nifedipine complex with GA at all dissociation states of GA. However, optical absorption experiments show the decrease of complex stability and solubility at pH > 6 when the GA molecule is fully deprotonated. It means the higher release rate of the drug in a neutral and basic environment compared with acid media. These results could form the basis of follow-up studies of GA self-associates as pH-controlled drug delivery systems.

## 1. Introduction

Encapsulation of lipophilic drugs with different delivery systems is an innovative approach that increases their solubility, stability, and bioavailability. Another advantage of the encapsulation of drug molecules into nanosized delivery systems is their controlled release in the human body [1]. Various delivery systems, such as inclusion complexes, micelles, nanoemulsions, nanoliposomes, and biopolymeric nanoparticles have been tested to improve drug properties. The basic concepts of nanoencapsulation are discussed in a number of recent reviews [2,3,4,5]. These reviews show the potential of nanoencapsulation technology and cover the main encapsulation methods.

Drug delivery systems can be divided into different groups according to the method of drug release: targeted drug delivery systems, controlled drug delivery systems, and modulated drug delivery systems. To date, pH and temperature-controlled drug releases are two approaches that can be used in medicine without a complicated control system.

Targeting of drugs is a significant aspect of drug delivery systems. It is classified into active and passive. Passive targeting works through a permeability increase, while active targeting is connected with the functionalization of drug delivery systems by specific ligands that interact with the receptors of certain cell types. For all targeting types, drug release can be triggered by a change in pH, temperature, or a combination of both. The main targets in the body are the receptors on cell membranes, lipid components of the cell membrane, and antigens or proteins on the cell surfaces [6].

The absorption of an orally administrated drug is influenced by different factors (drug stability and solubility, the activity of metabolic enzymes and drug transporters, etc.). It is known that pH values are different for different parts of an organism (gastrointestinal tract, different organs, tissues, and cellular compartments). For example, the pH value of the stomach is 1.5–3.5; pH values of the small intestine vary in the 5.5–6.8 range; pH values of the colon lie between 6.4 and 7 [7]. The pH values for cancer tissues are lower than for normal tissues, and the pH values of some cellular environments could alter from others (e. g., pH of endosomes is 5.5–6.0; pH of lysosomes is 4.5-5.0) [8]. For this reason, the pH-dependent behavior of a drug plays an important role in its functioning in the body. The use of pH-controlled drug delivery systems (DDS) opens a way to control drug release and target drug delivery when the drug is released only in tissues with specific pH values.

**Figure 1:**
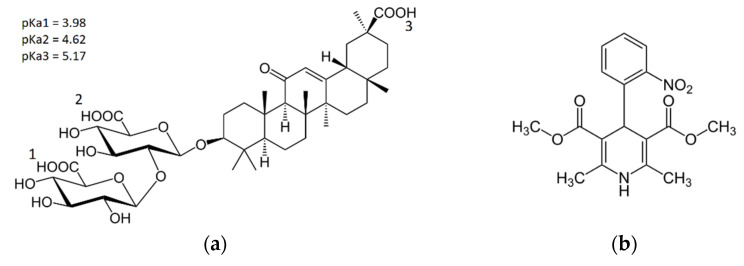
The chemical structure of (**a**) glycyrrhizic acid (GA) and (**b**) nifedipine.

Glycyrrhizic acid (glycyrrhizin, GA, Figure 1a) is the saponin from licorice root. Glycyrrhizin has been used in traditional Chinese and Japanese medicine since ancient times. It is widely investigated from the point of its biological activity: hepatoprotective, antiviral (including recently discovered activity against SARS-CoV-2), anticancer, etc. [9,10,11,12,13]. GA also demonstrates the ability to enhance the activity of other drugs [14,15,16,17,18,19,20,21]. In particular, it can reduce the toxicity, dose, and side effects of drugs and increase the therapeutic index [14]. This effect of glycyrrhizin has been observed, in particular, for nifedipine (NF), hypotensive, and antiarrhythmic drugs. Nifedipine complexes with GA manifested an antihypertensive therapeutic effect in a ten-fold reduced dose of nifedipine [14]. Taking into account the data indicating the decrease in the heart pressure under the use of GA [22], one can expect the synergistic effect of using the GA-NF complex. Several mechanisms for the biological activity of GA are proposed, and one of them is connected with the ability of GA to interact with cell membranes and to change membrane properties [23,24,25,26,27,28,29,30,31]. However, the explanation of the broad spectrum of the biological activity for GA is still under discussion.

It is known that glycyrrhizin forms self-associates (oligomers and micelles) in aqueous solutions, and its self-association depends on the pH of the media [32,33,34]. GA has three dissociation steps, with pKa values 3.98, 4.62, and 5.17 (Figure 1a, data is taken from [35]). Deprotonation of GA leads to changes in critical micelle concentration (CMC) values [32,33]. In addition, deprotonation leads to changes in the interactions of GA with lipid bilayers [33]. Therefore, such pH-dependent behavior suggests a possible application of GA as the base of a pH-controlled drug delivery system. In the present work, the properties of supramolecular associates of GA with a drug on the model of the hypotensive and antiarrhythmic drug nifedipine (Figure 1b) have been studied by the methods of nuclear magnetic resonance and molecular dynamics simulations. Earlier, it was found in vivo, that the complexes of GA with nifedipine show a significant increase (more than one order) in its therapeutic activity on the models of adrenaline-induced hypertension and CaCl_2_-induced arrhythmia [36].

## 2. Results

### 2.1. Optical Spectroscopy

**Figure 2:**
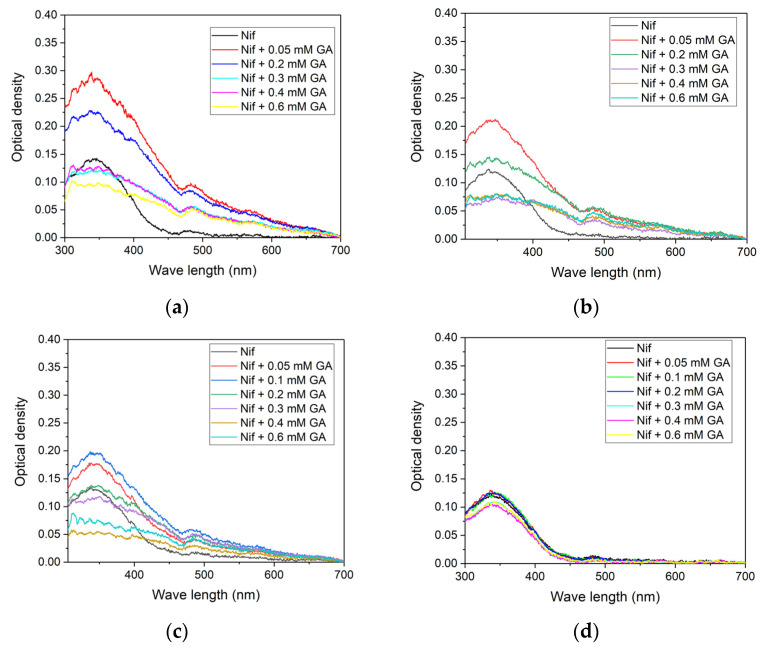
Optical absorption spectra of the mixture nifedipine + GA at pH value (**a**) 3.8, (**b**) 4.2, (**c**) 4.9, and (**d**) 9.5. The GA absorption is subtracted from all spectra. Nifedipine was added in an amount exceeding the maximum solubility in water (0.5 mM was added).

Optical absorption spectra of the mixtures of nifedipine with GA for different pH values are given in Figure 2.

**Figure 3:**
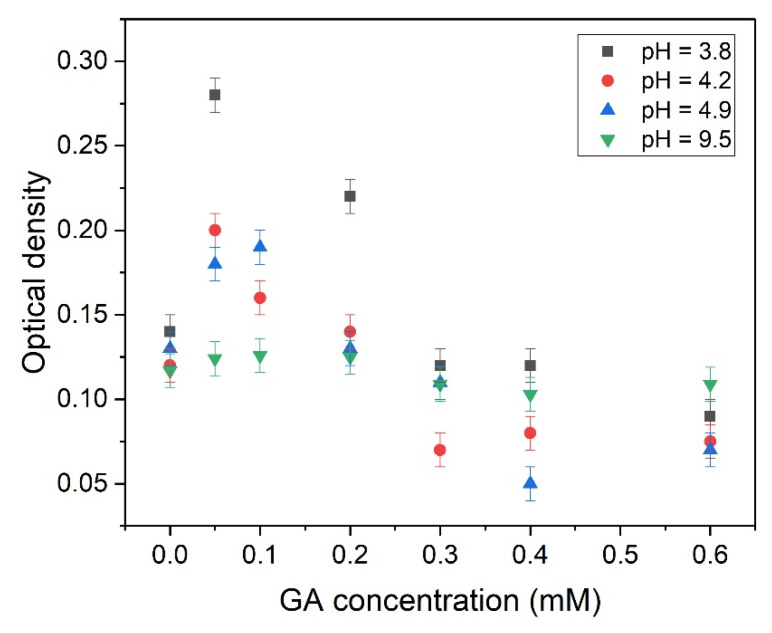
The dependence of the optical density of a mixture of nifedipine + GA on the concentration of GA at different pH values (3.8, 4.2, 4.9, and 9.5) at 342 nm.

The pH values of samples were chosen to achieve different degrees of dissociation of GA: pH 3.8 corresponds to fully protonated state, pH 4.2 corresponds to once deprotonated state, 4.9 corresponds to twice deprotonated state, and 9.5 (more than last pKa3 = 5.18) corresponds to fully deprotonated state (see Figure 1). The optical density in the absorption maximum of nifedipine (342 nm) with different concentrations of GA was measured for all series. The result is given in Figure 3.

It is noticeable that the concentration dependence of optical density demonstrates a similar behavior for all pH values: the increase of optical density at low GA concentration and the following decrease of optical density with the further increase of GA concentration (after the concentration of GA of about 0.1 mM). Supposedly, at low concentrations, GA could form complexes with nifedipine, which improves the solubility of the drug, which then leads to the increase of observed optical density. However, the further increase of GA concentration could lead to the formation of large insoluble aggregates and a decrease in the optical density. Similar behavior of nifedipine solubility on GA concentration was earlier detected in the water–methanol solution using an NMR technique [37]. The stoichiometry of the nifedipine-GA inclusion complex below critical micelle concentration (~1 mM) was calculated as 1:2 using Job plot measurement [37]. Besides, it is noticeable that at pH value 9.5 (a fully dissociated form of GA), the increase of optical density is absent. It could mean that the complex of nifedipine with GA at this pH is unstable.

**Figure 4:**
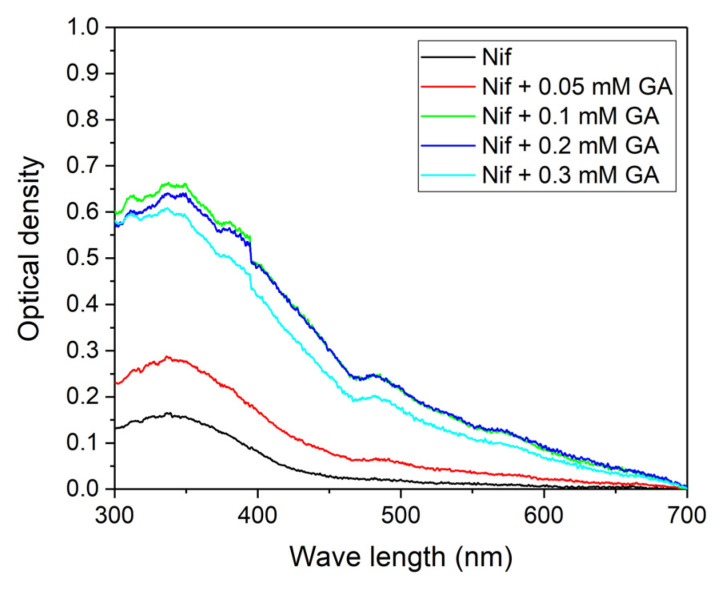
Optical absorption spectra of the mixture nifedipine + GA at pH value 4.9 after 24 hours of stirring. The GA absorption is subtracted from all spectra.

Longer stirring (24 hours) leads to a more significant increase in the optical density of nifedipine at low GA concentrations (Figure 4). An approximately six-fold increase of optical density was observed for pH 4.9. It can be suggested that due to the low solubility of nifedipine, complex formation with GA occurs slowly, which is why longer stirring improves the solubility of the complex.

### 2.2. Nuclear Magnetic Resonance Experiments

**Figure 5:**
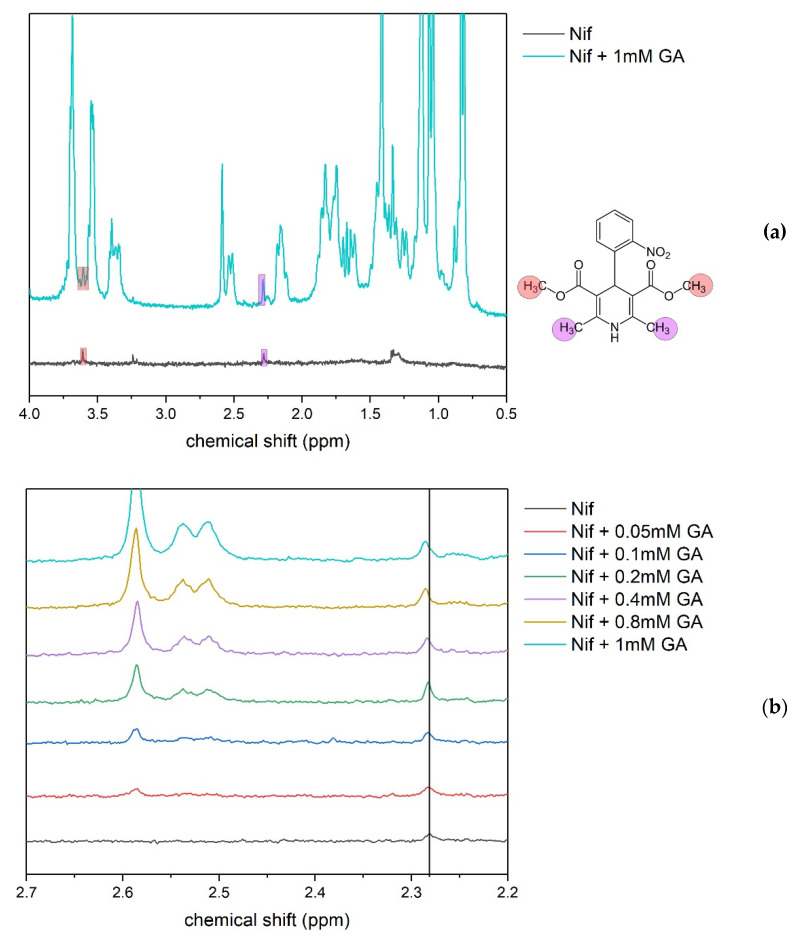
Fragments of 1H NMR spectra of mixtures nifedipine + GA at pH 6 (**a**,**b**). The assignment of nifedipine signals is taken from [38].

Fragments of ^1^H-NMR spectra of mixtures of nifedipine + GA with different concentrations at pH = 6 are shown in Figure 5a. Signals from aromatic protons are not observed, possibly due to the strong line broadening in the complex with GA. However, the shift of nifedipine signals corresponding to CH3 groups under the increase of GA concentration was detected (Figure 5b). This shift could be caused by the change of the nifedipine environment due to the formation of complexes with GA. It means that even at pH = 6, when the GA molecule is fully deprotonated, the complex with nifedipine is also formed.

The ability of GA molecules to form self-associates in an aqueous solution at different pH has been studied earlier by Petrova et al., using the NMR relaxation technique [25]. However, no data are available on the pH behavior of the inclusion complexes of glycyrrhizin with drug molecules. NMR diffusion measurements have been applied to study the behavior of the nifedipine-GA complexes at different dissociation degrees of the GA molecule in aqueous solutions. NMR diffusion experiments were done for samples with pH values 3.8, 4.2, 5, 6.1, and GA concentrations of 1mM. Diffusion coefficients of GA protons are given in Table 1. Under acidic pH, GA in protonated and partially deprotonated states could form micelles with CMC dependent on the pH value [32,39]. In diffusion NMR experiments, we studied the behavior of the aggregates with a GA concentration close to CMC.

As could be seen from Table 1, the diffusion coefficient demonstrates non-linear dependence on the pH value.

The size of aggregates could be estimated from this data using the following equation:

(1) $$D=\frac{\mathrm{kT}}{6\pi a\eta }$$

where D is a diffusion coefficient; T is a temperature; a is a radius; η is a viscosity; k is the Boltzmann constant. 

Using the data from Table 1, the radius of the GA-nifedipine inclusion complex is 1.3 nm. Larger aggregates (micelles) provide an impact on the observed NMR signal, but their impact on the diffusion coefficient is relatively small. Note that the size of GA micelles with incorporated drug molecules have been estimated in our earlier studies by the methods of gel permeation chromatography, dynamic light scattering, and electron microscopy. These experiments show that the size of GA micelles is in the range of 40–100 nm [40,41].

The increase of the diffusion coefficient with the increase of pH could be connected with the increase of CMC and, correspondingly, a decrease of the observed mean size of aggregates. However, the decrease of D value is observed at pH 6.1. At this pH, GA is in fully dissociated form, and such a decrease could not be connected with the micelle formation. Possibly, the changes in the structure of the nifedipine-GA complex, caused by Coulomb repulsion, could occur under these conditions, which leads to an increase in the radius of the associate. To answer this question, in this study, we have performed the molecular dynamics simulation of nifedipine-glycyrrhizin interaction at different dissociation degrees of GA molecule in an aqueous solution.

### 2.3. Molecular Dynamics Simulation

**Figure 6:**
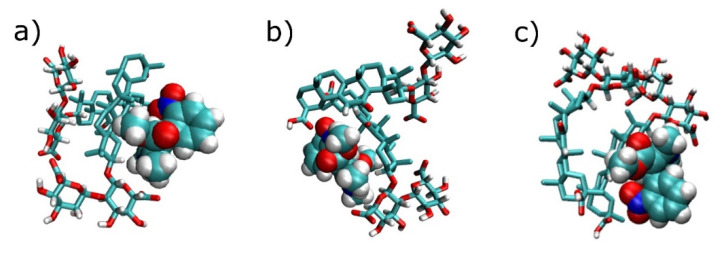
Examples of the complexes formed by two GA^2−^ ions and a nifedipine molecule in water (**a**–**c**). Nifedipine molecule is depicted by its van der Waals radii. Water molecules and Na ions are not shown.

To demonstrate the ability of GA at different dissociation degrees to form complexes with nifedipine, the behavior of two equally charged GA ions and a nifedipine molecule in water have been investigated. As it was noted above, 2:1 is the correct stoichiometry for the GA-nifedipine complexes at low GA concentrations [37]. For all dissociation steps of GA, the formation of stable complexes with nifedipine, which did not fall apart during the simulation run (200 ns), was observed. Examples of these complexes for GA^2−^ ion are shown in Figure 6. Apparently, same as neutral GA molecules, GA ions can form stable complexes with nifedipine in water, which can increase their solubility and facilitate its transport to and through a cellular membrane. A similar effect of GA has been recently shown for praziquantel [42].

**Figure 7:**
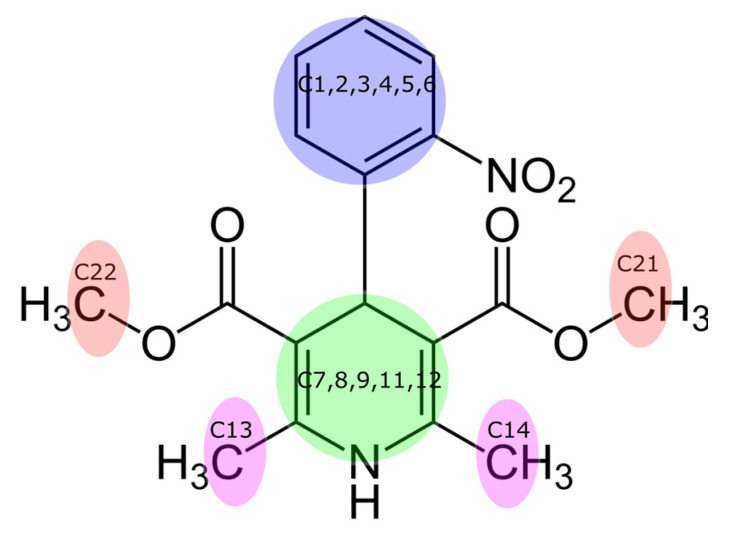
Moieties of a nifedipine molecule, which were used to measure distances from C13, C14 and C21, C22 carbon atoms of methyl groups, the pyridine ring and benzene ring are highlighted by magenta, red, green, and blue, respectively.

To characterize observed complexes, the distances between different moieties of the nifedipine molecule and the centers of mass (COM) of GA ions per simulation time were measured. These moieties and their color codes are shown in Figure 7.

**Figure 8:**
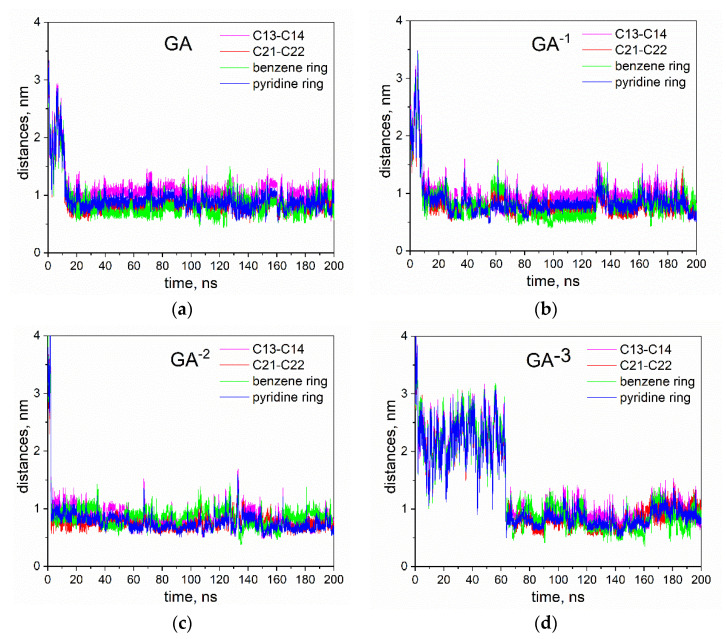
Distances between different moieties of the nifedipine molecule and the COM of different GA ions per simulation time (**a**) GA (fully protonated), (**b**) GA^1−^, (**c**) GA^2−^, (**d**) GA^2−^. Colors for different moieties are taken from Figure 7.

Figure 8 demonstrates the distances between different moieties of the nifedipine molecule and the COM of GA ions. The distances were calculated to the COM of both GA ions separately and then averaged. The distances from symmetrical C-atoms (C13 and C14, C21 and C22, Figure 7) were averaged too. The magenta line shows the averaged distance from C13 and C14 carbon atoms to the COM of GA ion. The red line depicts the distance from C21 and C22 atoms to the COM of GA ion, distances from the COM of the pyridine ring, and from the benzene ring are shown by green and blue, respectively. Distances were calculated for the system with neutral GA molecules and for ones with ions GA^1−^, GA^2−^, GA^3−^.

For all investigated systems, the formation of the complex of a nifedipine molecule and both GA molecules/ions (sharp decrease for all distances depicts the formation of the complex) was observed, and these complexes were retained during simulation runs.

Table 2 contains the distances from different moieties of the nifedipine molecule to the COM of GA, averaged over the last 100 ns of simulation time (in all runs, the molecules were in the complex during this period) and their standard deviation. The table also includes the distances between the centers of mass of the nifedipine molecule and GA.

Although the measured distances have rather large variances caused by the relative movement of the molecules in the complexes, from Figure 8 and Table 2, one can see particular structural patterns. In the case of neutral GA molecules, the average distance from the C13-C14 carbons of the methyl groups (attached to the pyridine ring) to the center of mass of the GA, is noticeably larger than the distances from other groups of nifedipine to the GA COM. This is clearly seen in Figure 8. Almost all the time the molecules were in the complex (20–200 ns), the magenta line is higher than others. At the same time, the distance between the COMs of the nifedipine and the GA molecule is the shortest. Similar correlations retain for the complex with GA^1−^ ion and are absent in the case of GA^2−^ and GA^3−^ ions. Note also that the distances for the complexes with GA ions, especially with GA^3−^ ion, have larger standard deviances. We believe that it can be the evidence of a less regular and possibly less stable structure of the complexes of nifedipine with GA ions compared with ones for neutral GA molecules. However, we highlight that all observed complexes both for GA molecules and GA ions were retained during simulations.

## 3. Materials and Methods

### 3.1. Materials

Nifedipine (1,4-dihydro-2,6-dimethyl-4-(2′-nitrophenyl)-3,5-pyridinedicarboxylic acid dimethyl ester, Sigma-Aldrich, St. Louis, MO, USA) and Glycyrrhizic acid (GA), a saponin from Licorice root (98%, Shaanxi Pioneer Biotech, Shaanxi, China), were used as supplied. ^1^H-NMR measurement spectra were performed in a D_2_O (Sigma-Aldrich, 99.9%) solution.

### 3.2. Optical Spectroscopy

Four series of samples with different pH values (3.8, 4.2, 4.9, and 9.5) were prepared. GA concentration in each series was varied in the range 0–0.6 mM. Nifedipine was added in an amount exceeding the maximum solubility in water (0.5 mM was added). All samples were stirred for 1.5 hours before measurement. Measurements were done on spectrophotometer SF-2000 (Spectrum, Russia) in a 1 cm quartz cuvette.

### 3.3. NMR Experiments

Series of samples with pH 6 was prepared for studying the possibility of a complex formation under GA dissociation. Nifedipine (2 mM) and GA with concentrations in the range 0–1 mM were placed in D2O with an amount of KOD that was necessary to achieve pH = 6. Samples were stirred for 1.5 hours before measurement. Diffusion measurements were done for pH values 3.8, 4.2, 5, and 6.1. All NMR measurements were made on Bruker AVANCE III (500MHz) spectrometer (Billerica, MA, USA).

### 3.4. Molecular Dynamics Simulations

The simulations were performed using the Gromacs 5.0.7 package and GROMOS54a7 force field (University of Groningen, Groningen, Netherlands). Topologies of GA ions with different dissociation steps GA^1−^, GA^2−^, and GA^3−^ were obtained using the Automated Topology Builder [43]. GA dissociation steps and corresponding constants pK are shown in Figure 1. The data are taken from [35]. Topologies of GA and nifedipine (NF) molecules were taken from the Automated Topology Builder (ATB) database. SPC water model was used. Each molecular dynamics (MD) model contained the number of Na ions needed to neutralize the total system charge. The simulations were performed in the NPT ensemble. Pressure (1 bar) was maintained by the isotropic Parrinello–Rahman barostat [44]. Constant temperature T = 310 K was maintained using Nose–Hoover thermostat [45] with the relaxation time of 2 ps. For electrostatic interactions, the Particle Mesh Ewald (PME) method with the fourth-order of cubic interpolation and the grid of 0.16 was used [46]. Initial configurations of the system represent two GA ions of equal dissociation step or neutral molecules and a nifedipine molecule located separately and surrounded by water (~10,000 water molecules). System size was about 7 × 7 × 7 nm. One production run of 200 ns duration for each dissociation step of GA (GA, GA^1−^, GA^2−^, GA^3−^) was calculated.

## 4. Conclusions

Complex formation of the hypotensive drug nifedipine with glycyrrhizic acid was observed at different pH values, corresponding to the different degrees of GA dissociation, including a fully dissociated state of GA. However, optical absorption experiments show that the increase of nifedipine solubility in the mixture with GA is significantly lower when the GA is fully deprotonated. Besides, the results of the measurements of the diffusion coefficient of GA indicate a change in the structure of the complex when GA is fully deprotonated. Such results could mean the changes in the structure of the nifedipine-GA complex in the solution with pH > 6 and a decrease of complex stability. It means the higher release rate of the drug in a neutral and basic environment compared with acid media. The radius of the GA-nifedipine inclusion complex estimated from diffusion experiments is equal to 1.3 nm. This value is in agreement with molecular dynamics simulation data.

In the present paper, the pH-sensitivity of the structure and stability of GA-nifedipine complexes have been demonstrated. For practical use, it means that the complex preparation should be done in acidic media, but drug release will be more effective in the neutral or slightly alkaline media. It could be connected with the decrease of the complex stability due to Coulomb repulsion. These results could form the basis of follow-up studies of GA self-associates as pH-controlled drug delivery systems.

## Figures and Tables

**Table 1:** Diffusion coefficients (D) of GA protons in the mixtures of nifedipine + GA for samples with different pH and 1mM of GA.

pH	D, *10^−10^ m^2^/s
3.8	1.9 ± 0.3
4.2	2.4 ± 0.1
5	2.7 ± 0.1
6.1	2.3 ± 0.1

**Table 2:** Distances (nm) between different groups of the nifedipine molecule and the center of mass (COM) of GA ions obtained from Figure 4, averaged over the last 100 ns of simulation time.

GA Ionization Form	COM of GA—C13-14	COM of GA—C21-22	COM of GA—Benzene Ring	COM of GA—Pyridine Ring	COM of GA—COM of Nifedipine
GA	0.99 ± 0.14	0.80 ± 0.11	0.83 ± 0.15	0.86 ± 0.12	0.78 ± 0.10
GA^1^^−^	0.95 ± 0.14	0.76 ± 0.12	0.78 ± 0.18	0.82 ± 0.12	0.74 ± 0.12
GA^2^^−^	0.79 ± 0.15	0.77 ± 0.12	0.84 ± 0.16	0.73 ± 0.12	0.73 ± 0.11
GA^3^^−^	0.88 ± 0.21	0.79 ± 0.23	0.79 ± 0.19	0.84 ± 0.15	0.78 ± 0.15

## Data Availability

The data presented in this study are available on request from the corresponding author.

## Sample Availability

Samples of the compounds are not available from the authors.

## Abbreviations

CMC	critical micelle concentration
COM	center of mass
DDS	drug delivery system
GA	glycyrrhizic acid
MD	molecular dynamics
NF	nifedipine
NMR	nuclear magnetic resonance
